# Evaluating Housing Health Hazards: Prevalence, Practices and Priorities in Delhi’s Informal Settlements

**DOI:** 10.1007/s11524-020-00442-w

**Published:** 2020-05-30

**Authors:** Emily Nix, Jacob Paulose, Clive Shrubsole, Hector Altamirano-Medina, Michael Davies, Renu Khosla, Kristine Belesova, Paul Wilkinson

**Affiliations:** 1 UCL Institute for Environmental Design and Engineering, Bartlett School of Environment, Energy and Resources University, University College London, Central House, 14 Upper Worburn Place, London, WC1H 0NN UK; 2 Centre for Urban and Regional Excellence, 4, Second Floor, Zamrudpur Commercial Complex, Greater Kailash, New Delhi, 110048 India; 3 Air Quality and Public Health Group, Environmental Hazards and Emergencies Department, Centre for Radiation, Chemical and Environmental Hazards, Public Health England, Harwell Science and Innovation Campus, Oxfordshire, OX11 0RQ UK; 4 Centre on Climate Change and Planetary Health & Department of Public Health, Environments & Society, London School of Hygiene & Tropical Medicine, 15-17 Tavistock Place, London, WC1H 9SH UK

**Keywords:** Housing, Health hazards, Participatory approach, Informal settlements, Community priorities

## Abstract

Housing quality is crucially linked to health and sustainability goals, yet there is limited research on informal housing and settlements where housing quality is poor, and the health risks are expected to be greatest. This paper describes the investigation of housing conditions in a low-income resettlement colony in Delhi. A novel transdisciplinary methodology to evaluate multiple housing health hazards and establish intervention priorities in participation with the community was developed. Findings from housing surveys and indoor environmental monitoring were contrasted with a participatory self-assessment—revealing the widespread prevalence of hazards and suboptimal housing conditions as well as substantial differences in priorities, and thus perspectives, between participants and researchers. Focus group discussions explored the findings and built consensus on priorities. Our findings uncovered how poor housing conditions affect daily practices and thus are likely to adversely affect socio-economic development and gender equality. We highlight limitations in current frameworks to assess housing hazards and argue that a transdisciplinary approach is vital to provide a holistic understanding and to develop effective interventions. These insights are crucial to inform inclusive solutions for adequate housing and human settlements that can support improved health and help achieve the sustainable development goals.

**Electronic supplementary material:**

The online version of this article (10.1007/s11524-020-00442-w) contains supplementary material, which is available to authorized users.

## Introduction

Access to adequate housing is a fundamental human right [1], and achieving this is crucial to realising the United Nations Sustainable Development Goal (SDGs) 11 to “make cities and human settlements inclusive, safe, resilient and sustainable” [2]. Poor housing has appreciable health burdens via exposure to indoor temperatures [3]; pollutants [4]; dampness and mould [5]; injuries from falls, fires, and electrocution; ingress of disease vectors [6] and infectious diseases from inadequate household facilities [7, 8]. Research highlights these connections and indicates that housing improvements can not only improve health outcomes [9–12] but also help reduce health inequalities [13] and meet energy efficiency targets [14], helping to contribute to social, environmental and economic development goals [15].

Informal housing, which constitutes between 60 and 90% of housing in developing countries [16], falls outside formal planning regulations and is often of varied quality, failing to meet health and sustainability requirements. Housing conditions in informal (slum) settlements, in particular, are extremely poor, resulting in avoidable health hazards [17]. The global proportion of those living in slums is predicted to increase to 25% by 2030, yet there have been a limited focus on health [17] and little evidence on the health impacts of housing conditions and improvements in slums [18]. Additional data on multiple health risks in informal settlements is recognised as crucial in achieving 2030 SDGs [19]. Understanding current housing conditions is vital to inform interventions that can simultaneously contribute to enhanced health and sustainability.

Informal housing and slum growth have dominated Indian cities. In Delhi, 75% of housing is unplanned [20] and 1,020,423 households (approx. 30% [21]) are located in Delhi’s 6343 slums [22], which are predominately located on public land (78.25%) and have electricity (99%) access to water via a tap (87%) or hand pump (13%), but most have no sewer (84%) and tend to have open drains (91%) [22]. Housing is mainly built from solid “*pucca*” materials (55% have roof *and* walls made from solid materials and 30% either solid walls *or* a solid roof). Sanitation is mixed (16% use an owned (*assumed household*) pit latrine, 13% a shared service latrine, 29% a community flush/septic tank, 18% a community service latrine and 22% no latrine) [22]. Of children 0–5 years, 8.3% had a fever, 4.9% had symptoms of acute respiratory infection and 8.3% had diarrhoea. This suggests varied infrastructure and potential health risks [23], although data between surveys tend to differ (for example between [22] and [23]) and should be treated with caution. Details on water source, sanitation systems and principle housing materials are useful, but data is often unclear and cannot be translated into health indicators. Further work is vital to understand the multitude of risks to health in these settings.

This research assessed housing health hazards and prioritises interventions to support the development of solutions in an informal settlement in Delhi. It forms part of a research project “Optimising housing for health and sustainability goals in low-income settings (Optihouse)”, which investigates how housing improvements can contribute to health and sustainability goals [24]. It uses a participatory action research (PAR) methodology bringing together academics, the community and local development practitioners to develop housing solutions that are locally sustainable and scalable. This paper discusses the results obtained during the problem identification phase of the work [24], where the objectives were to (1) assess current housing conditions and prevalence of housing health hazards; (2) understand the households’ perceptions of hazards and their lived experiences and (3) build consensus with the households on priorities for interventions.

## Methods for Assessing Housing Health Hazards

There have been several assessment frameworks developed to assess housing hazards and evaluate the likely level of harm to health in high-income countries [25]. There are no widely established standardised frameworks: with approaches varying between countries and in their purposes [26]. New Zealand’s Healthy Homes Index assesses hazards through visual inspections and measurement of housing characteristics [27]. Similarly, the UK Housing Health and Safety Rating System (HHSRS) uses housing inspections to score hazards, with ratings based on population health data for typical UK homes [28]. Other frameworks rely solely on visual household inspections [8, 29], while others used sampling to determine contamination levels [8]. There is no alignment between existing frameworks, with the hazards included and the assessment methods varying depending on the context. These frameworks are unsuitable for direct use in low-income settings, as the hazards present and their recommended assessment differ significantly. More advanced methods that rely on linking detailed data on housing and health are limited or non-existent in low-income settings.

Current frameworks employ quantitative approaches and do not consider socio-economic impacts and connections between housing and health. Consequently, they fail to recognise interactions between housing hazards and household practices. For example, through measuring indoor temperatures, heat exposure can be recorded and judged. However, by understanding what behaviours take place (such as whether cooling appliances are used), it is possible to develop a better understanding of the exposure to harm which can help design more effective intervention responses. The methodology used in this work attempts to overcome these issues by using a transdisciplinary approach to assess housing conditions and evaluate hazards.

## Methodology

### Case Study

**Fig. 1:**
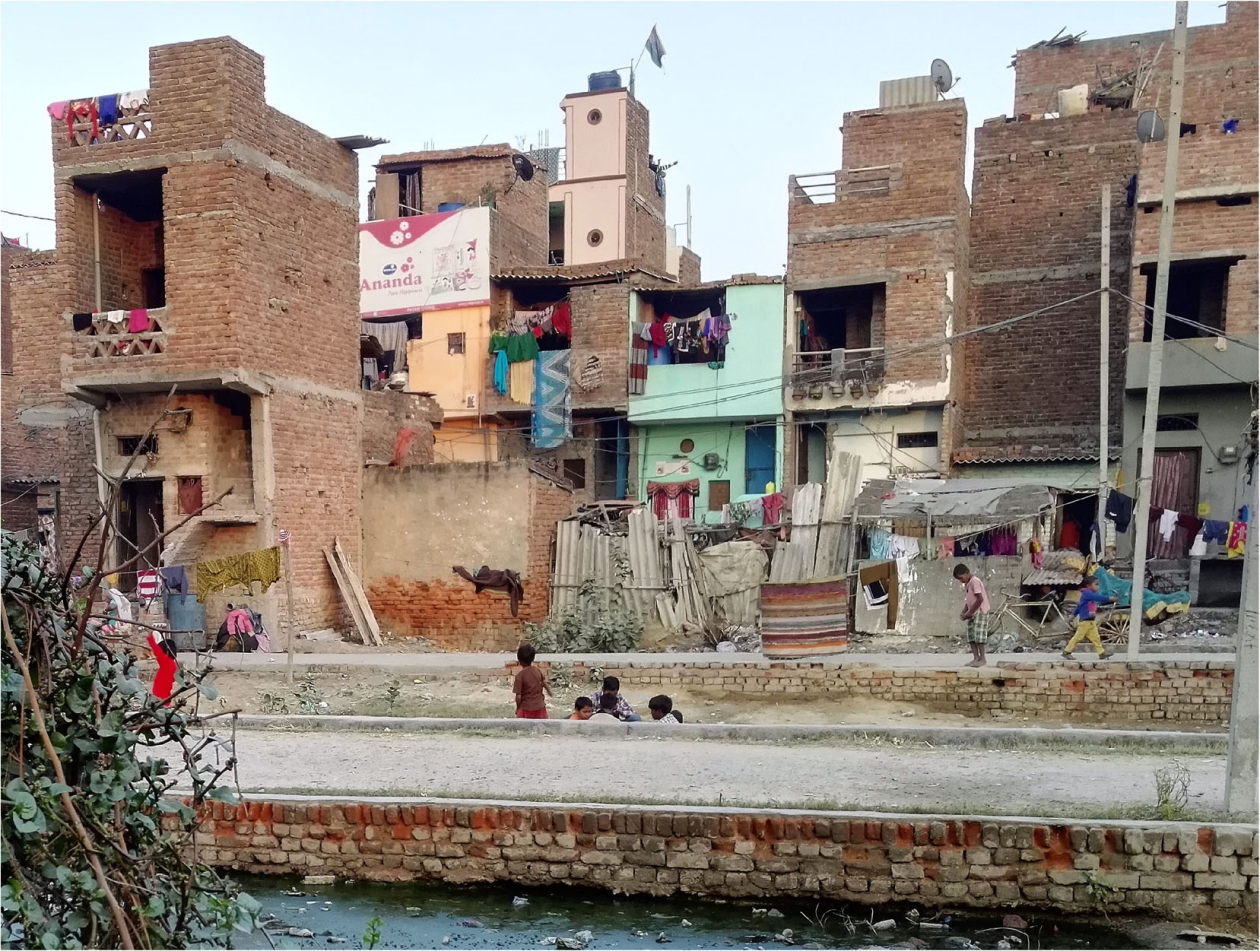
A typical street and range of housing typologies in Savda Ghevra

The resettlement colony, Savda Ghevra, located on the North-West edge of Delhi was selected as the case study. The settlement is home to around 7000 families relocated from inner-city slum areas, where small empty plots of 12.5 m^2^ or 18 m^2^ were provided for independent construction on 10-year leases. Currently, it is unclear what will happen after the tenure expires. Plots are arranged in back-to-back rows, and each block has an open space designed as a park and community sanitation block. A typical street and the dwellings can be seen in Fig. 1. There is no piped water or sewage connection, with treated water delivered daily by tankers.

Dwellings range from one-storey “*kutcha*” constructions to established four-storey “*pucca*” constructions with roof space and toilet^1^ (Table 1). The building process is incremental and relies on available skills and resources, with little or no external assistance. Twenty-seven households were recruited to participate through a series of community workshops that introduced the project aims. Participation was based on willingness, but representativeness of the different typologies was encouraged. Descriptions of each typology surveyed are detailed in Table 1 (see images in the Supplement Material (SM) provided by the authors). At least one representative from each household took part in the research—most often a female. Female occupants are most likely to be affected by inadequate housing, as they often stay at home throughout the day and are responsible for most household tasks. This is particularly the case in India, where gender inequality is well reported.

**Table 1:** Participating households by typology as found in the resettlement colony

Typology	Description	Number of households
Kutcha	Single storey. Construction from temporary materials (plastic sheeting, bamboo etc.). No toilet.	4
Semi-pucca	Single storey. Brick walls, cement floor, and corrugated roof. No toilet.	5
Pucca 1	Single storey. Brick walls, cement floor, and stone or concrete roof. No toilet.	2
Pucca 1.5	Single storey with roof space. Brick walls, cement floor, and stone or concrete roof. Toilet.	6
Pucca 2	Two storeys. Brick walls, cement floor, and stone or concrete roof. Toilet.	5
Pucca 2.5	Two storeys with roof space. Brick walls, cement floor, and stone or concrete roof. Toilet.	2
Pucca 3+	Three storeys plus. Brick walls, cement floor, and stone or concrete roof. Toilet.	3

### Research Methods

A transdisciplinary mixed-method approach was developed to gather and analyse evidence on the housing conditions. This drew on disciplines and methodologies from the built environment, health, social science and development studies, and included community members in defining research objectives. A broad risk assessment framework of identification, analysis and evaluation was followed [30] (see SM). Dwelling surveys captured evidence on housing conditions, occupant surveys captured perceived comfort and behaviour, and indoor environmental monitoring captured indoor exposure levels. This evidence was analysed by a risk assessment to score and rank hazards. To understand the residents’ perspective, residents ranked risks using a set of hazard picture cards. Follow-up focus group discussions then evaluated the findings and built consensus on the key problems. Transect walks followed the focus groups to investigate the key cause of prioritised hazards.

### Selection of Hazards

The first step involved the selection of hazards; these were derived from the existing housing hazard frameworks (see the “Methods for Assessing Housing Health Hazards” section) and refined through discussion between the research team. Some hazards were simplified; for example, indoor air pollution was included as a single category rather separate pollutants. The final list of 21 hazards is included in Table 2.

**Table 2:** Method of identification and indicator for each household hazard

No.	Household hazard	Method of identification	Indicator
1	Damp and mould	Dwelling survey	Extent of mould on internal/external surfaces
2	Heat	IEQ monitoring/occupant survey	Recorded temperature and perceived comfort during summer
3	Cold	IEQ monitoring/occupant survey	Recorded temperature and perceived comfort during winter
4	Indoor air pollution	Dwelling survey/occupant survey	Location of cooking, ventilation provision and perceived air quality
5	Asbestos	Dwelling survey	Presence of asbestos
6	Overcrowding	Dwelling survey/occupant survey	Number of occupants in the given space
7	Security/intruders	Dwelling survey	Presence of locks and bars on openings
8	Inadequate lighting	IEQ monitoring/dwelling survey/occupant survey	Level of lighting (lux) and perceived lighting
9	Noise	Occupant survey	Perceived noise levels and building permeability
10	Mosquitoes	Dwelling survey	Presence of open water storage and drains
11	Domestic hygiene	Dwelling survey	Quality of kitchen facilities and location of drains
12	Pests	Dwelling survey	Presence of pests
13	Food safety/infestations	Dwelling survey	Presence of refrigerator
14	Sanitation and drainage	Dwelling survey	Quality of bathing facilities
15	Personal hygiene	Dwelling survey	Presence of toilet and sanitation system
16	Water supply	Dwelling survey	Water source type
17	Falls	Dwelling survey	Ergonomics of staircase, use of space and levelling of the floor
18	Electrical shocks	Dwelling survey	Quality of electrical fittings, exposed wires and proximity of water
19	Fire	Dwelling survey	Location of cooking area, cooking fuel used and quality
20	Collision and entrapment	Dwelling survey	Ergonomics of dwelling and space
21	Structural collapse	Dwelling survey	Quality of the dwelling structure

### Hazard Identification and Analysis

#### Survey-Based Risk Assessment

Hazards were identified through dwelling survey, occupant survey and indoor environmental monitoring. The dwelling survey, completed by trained architects and engineers, gathered data on dwelling characteristics, facilities and dwelling use, as well as recording measurements and producing architectural drawings. The occupant survey recorded residents’ perceptions, e.g. comfort during winter and summer, and their operation of the dwellings, e.g. use of extract fan and openings. Indoor environmental monitoring of temperature, relative humidity and lighting levels was carried out (10 min intervals over a year) to determine exposure to relevant hazards. The identification method and indicator used for each hazard were based on the expertise and resources (Table 2). For example, the survey recorded the water source used. For heat, the occupant survey recorded the perceived comfort level and environmental monitoring equipment captured the indoor temperatures.

Hazards were analysed using a semi-quantitative method based on the likelihood of occurrence and expected harm. A consequence/probability matrix rated risks: allowing for comparison between multiple household hazards. After evidence was compiled, the surveyor rated the likelihood of occurrence, based on a scale: low, moderate, high and severe based on frequency and magnitude of occurrence. Expected harm was rated between low and severe based on potential health outcomes.

#### Self-assessment

Picture cards with images representing hazards were developed. Images were screened for appropriateness by local field facilitators (see SM). Each household was introduced to the hazard cards and their relevance explained during community workshops. Households took the cards away to identify the hazards present in their houses.

Households were asked to rank hazard cards in order of priority during the survey visit. Researchers ensured that the participant understood the hazard depicted in each card during the ranking process and recorded the order of priority. Rankings were recorded and compiled to generate a self-assessment matrix.

### Hazard Evaluation

Six focus group discussions (FGDs) were held according to dwelling typology^2^, groups were semi-structured but had two distinct elements. The first focused on the findings from the self-assessment, where the households were asked about the occurrence of the reported hazards and impacts. The second focused on the survey-based risk assessment findings, where participants were asked about the impacts of these hazards. Participants were asked to prioritise hazards, and then, a discussion on potential conceptual solutions was held. An interpreter was present during the FGDs for the non-Hindi-speaking researchers. FGDs were recorded, translated and transcribed for analysis. Transect walks followed the FGDs and individual household visited to fully investigate the problems identified. Field notes and photos were taken. This allowed for triangulation and validation of the problems reported by the occupants.

Qualitative data from FGDs, field notes and photos were analysed using NVivo v12 Pro (QSR International) [31]. We followed the framework analysis method, developed for applied qualitative research [32] widely used in multi-disciplinary health research [33], which consists of five stages: familiarisation, identification of an analytical framework, indexing, charting, and mapping and interpretation. Two researchers read transcripts and listened to audio recordings to become familiar with the data. Open coding was completed to generate an initial set of codes. These key themes and related codes were refined through an iterative approach of recoding and discussions between the researchers, before an analytical framework and final code set were agreed. Indexing was completed by applying the developed framework to all the transcripts and data, after which the indexed data was charted by generating a matrix of cases (housing typologies) and codes for each of the key themes identified. Interpretation and mapping were guided by the original research objectives and carried out by reviewing the data contained in the matrices and making connections within and between codes and cases.

## Results

### Housing Conditions and Impacts on Health, Well-being and Daily Practices

Key characteristics of surveyed dwellings can be seen in Table 3. There was a high occurrence of hazards across the dwellings, with the temporary *kutcha* dwellings experiencing the highest prevalence of hazards and most limiting conditions; occupants did not recall any positive aspects of the house:

I don’t like anything about the house. We are just living there. Out of obligation. (FGD-3)

**Table 3:** Characteristics of the surveyed households

HH No.	Typology^i^	Dwelling age	Occupancy No.	Floor area (m^2^)	Toilet^ii^	Water supply	No. of rooms	Wall material	Roof material	Floor material	Cooking fuel	Air cooler	Structural quality^iii^	Airtightness^iii^	Electric quality^iii^	Mould presence^iv^
A	Kutcha	9	6	12.2	N	Tanker	1	Bamboo and plastic sheets	Bamboo, plastic sheets	Brick and render	LPG	N	P	P	A	W
B	Kutcha	10	9	11.9	N	Tanker	1	Brick	Wooden sheets	Mud	LPG	Y	P	P	P	W
C	Kutcha	10	6	10.5	N	Tanker	1	Brick and render	Corrugated	Plastic mats	Wood, cow dung	N	P	P	P	W
D	Kutcha	10	5	10.2	N	Shared borewell and tanker	1	Brick	Corrugated	Mud	LPG	N	P	A	P	W
E	Semi-pucca	7	4	25	Y	Borewell and tanker	2	Brick and render	Corrugated	Concrete	LPG	Y	G	G	P	W
F	Semi-pucca	10	6	10.2	N	Tanker	1	Brick and render	Corrugated	Concrete	LPG	N	P	A	P	W
G	Semi-pucca	10	3	10.4	Y	Borewell and tanker	1	Brick	Corrugated	Concrete	LPG	Y	A	A	P	-
H	Semi-pucca	5	5	10.1	N	Borewell and tanker	1	Brick and render	Corrugated	Concrete	LPG	Y	A	P	P	W
I	Semi-pucca	8	5	10.3	N	Tanker	1	Brick and render	Corrugated	Concrete	Wood and cow dung	N	P	P	P	W
J	Pucca1	10	6	10.4	Y	Borewell and tanker	1	Brick and render	Stone slabs	Concrete	LPG	Y	G	A	A	S
K	Pucca1	8	5	10.4	Y	Borewell and tanker	1	Brick and render	Stone slabs	Concrete	LPG	Y	G	A	P	-
L	Pucca1.5	10	3	24.3	Y	Borewell and tanker	2	Brick	Stone slabs, Corrugated	Concrete	LPG	Y	A	P	P	S
M	Pucca1.5	9	7	22.6	Y	Tanker	3	Brick and render	Stone slabs, Corrugated	Concrete	LPG	Y	G	P	P	S
N	Pucca1.5	3	7	24.3	Y	Shared borewell and tanker	3	Brick	RCC, Corrugated	Concrete	LPG	Y	P	G	P	W
O	Pucca1.5	8	11	27.7	Y	Borewell and tanker	3	Brick	RCC, Corrugated	Concrete	LPG, wood	N	A	A	P	-
P	Pucca1.5	8	5	23.1	Y	Borewell and tanker	4	Brick and render	Stone slabs, Corrugated	Concrete	LPG	Y	A	G	P	W
Q	Pucca1.5	4	5	23.5	N	Borewell and tanker	2	Brick and render	RCC, Corrugated	Concrete	LPG	N	G	G	G	W
R	Pucca2	10	8	37.6	Y	Borewell and tanker	5	Brick and render	RCC	Concrete	LPG	N	G	G	G	W
S	Pucca2	8	8	24.6	Y	Borewell and tanker	3	Brick	RCC	Concrete	LPG	Y	P	P	P	S
T	Pucca2	5	5	23.3	Y	Borewell and tanker	2	Brick	RCC	Concrete	LPG	Y	G	G	G	W
U	Pucca2	2	9	38.4	Y	Borewell and tanker	4	Brick and render	RCC, Stone slabs	Concrete	LPG	N	G	G	G	W
V	Pucca2*	4	3	53	Y	Borewell and tanker	6	Brick and render	RCC, Stone slabs	Concrete	LPG	Y	G	P	G	W
W	Pucca2.5	5	6	37.1	Y	Borewell and tanker	6	Brick and render	Stone slabs, Corrugated	Concrete	LPG	Y	G	G	P	W
X	Pucca2.5	9	1	27.5	Y	Tanker	3	Brick and render	RCC	Concrete	LPG	N	A	G	P	S
Y	Pucca3+	1	3	37.1	Y	Borewell and tanker	5	Brick, render and tiles	RCC	Concrete	LPG	Y	G	G	A	S
Z	Pucca3+	0.5	10	37.5	Y	Borewell and tanker	6	Brick and render	RCC	Concrete	LPG	Y	G	G	G	-
AA	Pucca3+	7	8	35.6	Y	Borewell and tanker	6	Brick, render and tiles	RCC	Concrete	LPG	Y	G	G	G	S

^i^Plot sizes are 12.50 m^2^, except for those indicated with an asterisk which have a plot size of 18.0 m^2^. ^ii^Y indicates yes and N indicates no. ^iii^P indicates poor, A—adequate and G—good. ^iv^S indicates some and W—widespread

In general, all dwellings were found to be inadequate with some level of hazard. Hazards were found to significantly influence the daily practices of participants who employed a wide range of coping strategies though some are likely to pose further health risks. A summary of the prevalence of each hazard and their impacts is detailed in Table 4.

**Table 4:** Summary of prevalence, the reported impacts and coping strategies for each hazard

	Household hazard	Prevalent conditions	Reported impacts	Coping strategies
1	Damp and mould	Widespread in 19 (of 27) dwellings, rising damp seen on walls. Flooding of dwellings during heavy rains	Damp bedding, leaks during the rainy period, damaged furniture, breathing problems and asthma	Floor covering, re-painting and plastering, tilling
2	Heat	Mean daily temperature above 29 °C in all dwellings during the hottest month (May)	Feeling of suffocation, difficulty sleeping without coolers, dizziness and nausea	Use of ground floor spaces, bathing 2–3 times a day, sleeping on the ground, use of fans, coolers
3	Cold	Mean daily temperature below 19 °C in all dwellings during the coldest month (January)	Feeling of discomfort	Use of blankets and shawls, sitting in sun, warming selves on the stove, burning of firewood indoors
4	Indoor air pollution	Cooking primarily with LPG, poor ventilation provision, where wood or cow dung was used, this was in the street	Coughing due to spices, smoke entering from nearby cooking	Opening of doors and use of extract fans in some dwellings (7 households)
5	Asbestos	Corrugated sheets widely used for roofs, which may contain asbestos	-	-
6	Overcrowding	Less than 5 m^2^ per occupant in 21 of the dwellings	18 reported having inadequate space, sharing single rooms and beds, lack of privacy	-
7	Security/intruders	No dwellings had bars on windows	Theft reported by two households	Locking of doors
8	Inadequate lighting	13/27 of dwellings reporting poor lighting, with a dependency on artificial lighting	No sunlight and not seeing when entering the dwellings	Light candles to cook, leave lights on due to the contrast between indoors and outdoors
9	Noise	17/27 of households finding it noisy and 12/27 having disturbed sleep due to noise	Lack of privacy and disturbance from neighbours	-
10	Mosquitoes	Mosquitoes widespread, open drains and water storage	Mosquitoes reported bothersome and continuously biting, sickness from dengue and chikungunya	Bed-nets, repellents, use of ceiling fans and cow dung to produce smoke
11	Domestic hygiene	14 of dwellings deemed poor, poor solid waste management	Multi-purpose space requires regular cleaning	Frequent cleaning
12	Pests	Rats and mice commonly seen, stray dogs and livestock present	Residents bitten by rats	Frequent cleaning, traps, blocking entry
13	Food safety/infestations	12/27 households owned refrigerators, inadequate storage in less than half of homes	Insects infested foods, skin irritation and rashes	Sorting and washing food to remove insects, preservatives,
14	Sanitation and drainage	19/27 of households had home toilets, open defecation widely practised, community sanitation blocks poorly maintained	Distress due to lack of toilet, dependency on neighbour	Reducing the frequency of defecation
15	Personal hygiene	Washing facilities poor in 19 of 27 homes	-	-
16	Water supply	Water from tankers, 20 households also using a borewell	-	-
17	Falls	Steep staircase on the outside of dwelling with no railings	-	-
18	Electrical shocks	Electrical quality recorded as poor in 17 of 27 households, the widespread use of immersion rods for water heating	Shocks reported, particularly in rainy season	-
19	Fire	Cooking in cramped location with retrofitted gas cylinders	-	-
20	Collision and entrapment	Small cramped spaces, narrow stairways and low head-clearance	-	-
21	Structural collapse	Majority of dwellings no columns, where they do these are not as per the required standard	Fear of dwelling collapsing during storms	

**Fig. 2:**
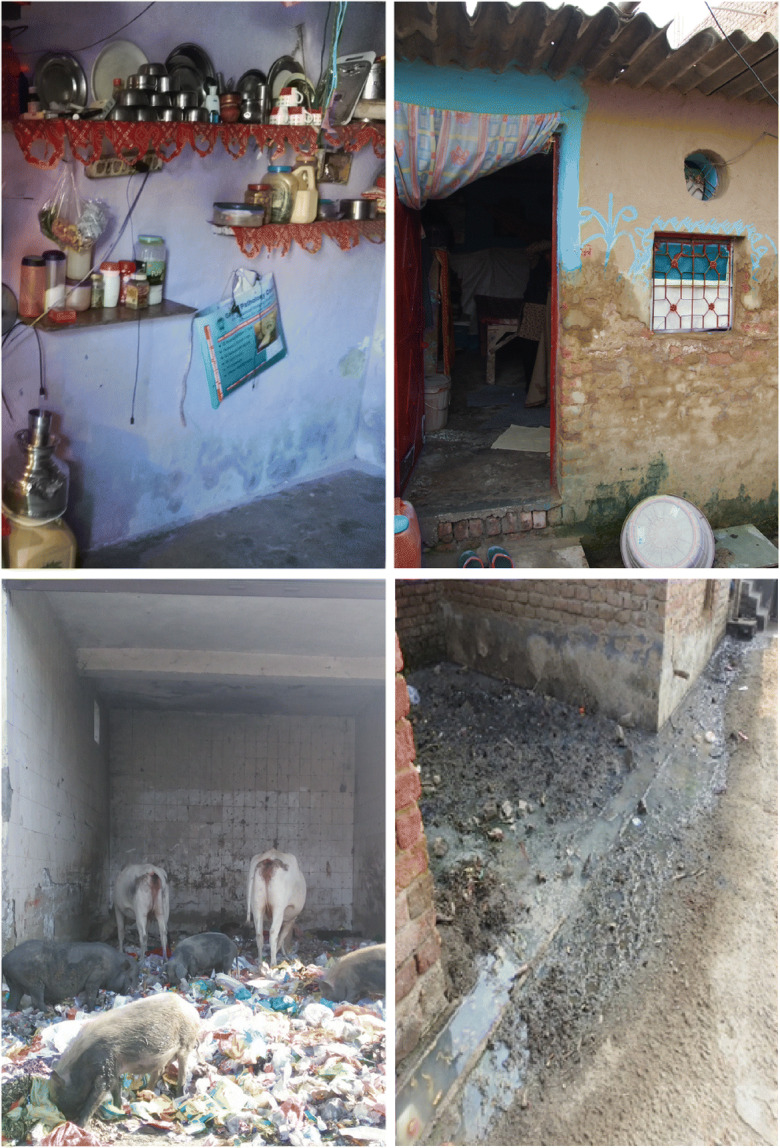
Top left: dampness at present at the lower part of the wall. Top right: practices of blocked window openings. Bottom left: solid waste with the presence of animals. Bottom right: open drains containing wastewater

Damp and mould were widespread in 16 of 27 houses (some damp in seven and none in four), predominantly at the lower part of the walls (Fig. 2). The focus group discussion revealed that flooding and leaks were extensive, particularly where dwelling entrances were beneath road level:

…when it rains the entire rainwater comes inside our house, we use buckets and throw the water out… (FGD-1)

Participants reported breathing problems, particularly asthma, were higher in damp conditions, with children experiencing coughs and cold. Households dealt with dampness by covering floors with “gunny” bags and tilling, re-painting with oil-based paints and plastering walls up to “every 2–3 months”; however, this was often ineffective as damp rose further. Participants recalled putting bedding out to dry every day after sleeping on damp floors, indicating significant time and effort coping with the hazards.

**Fig. 3:**
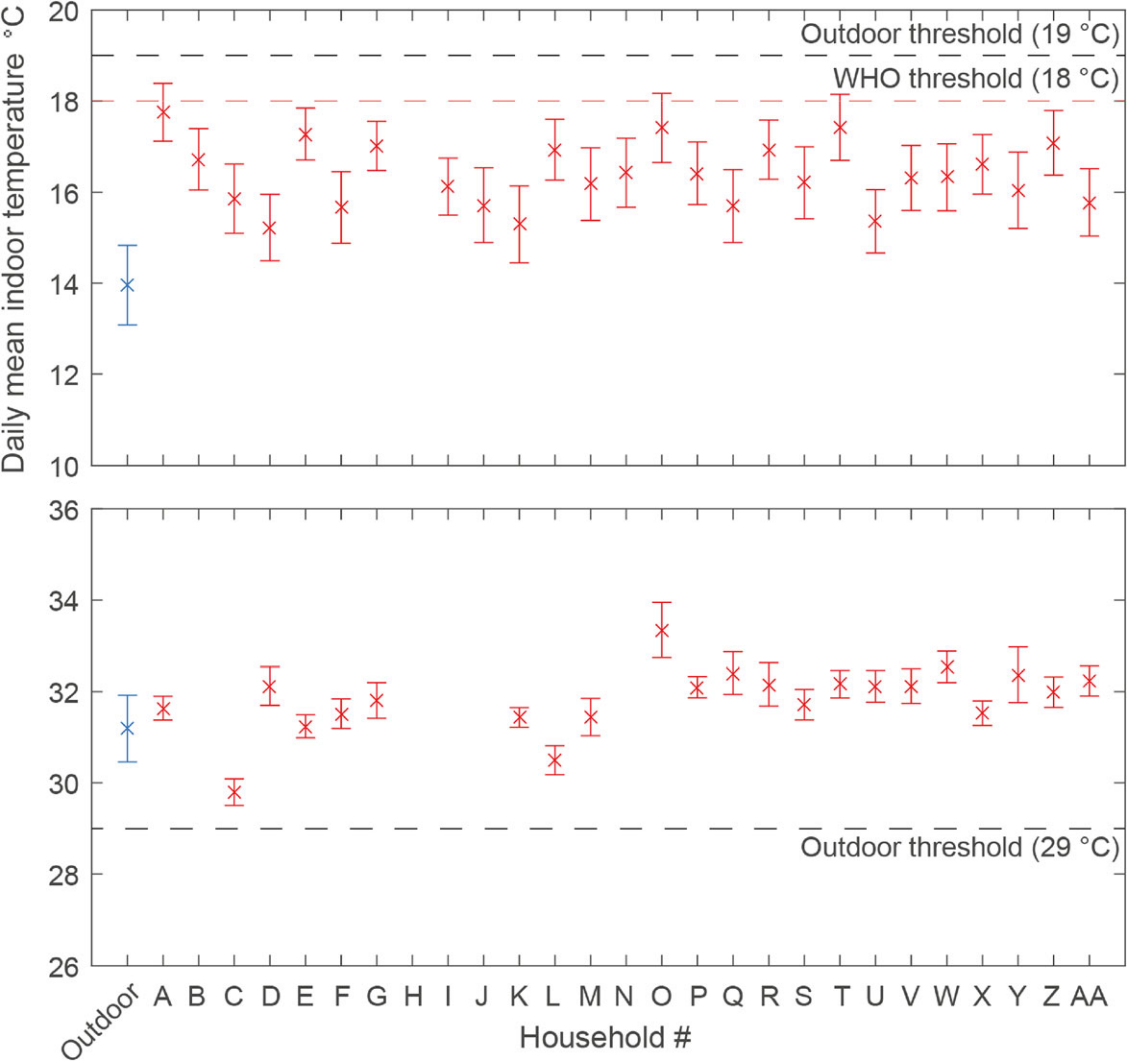
Average daily mean indoor temperature during January (top) and June (bottom) in surveyed households, plotted with 95% confidence intervals*.* For cold of 18 °C [7] or the outdoor cold-related mortality threshold of 19 °C [34]

During summer, the daily mean indoor temperature remained above the outdoor heat-related mortality threshold [34] (Fig. 3). This suggests poor thermal control and significant risk to health and comfort, although 17 houses owned air coolers and all dwellings had ceiling fans. The heat was described to be “nauseating” often resulting in occupants “feeling dizzy”, and having to lie down near a cooler. Both indoors and outdoors, the conditions were uncomfortable:

…because of the heat, we cannot stay indoors or outdoors… it is very hot outside, no trees, so we cannot sit outside, and inside also is very hot, it is very difficult for us (FGD-1)

To keep cool, occupants bathed “2–3 times” a day, occupied the cooler ground floor spaces, slept directly on the ground or outdoors (terrace or street), applied water or mud to the floor and used fans and air coolers to reduce temperatures.

During the coldest month, daily mean temperature indoor remained below the recommended guidance (Fig. 3), with up to 4 °C difference between dwellings. Airtightness was recorded to be poor in many dwellings, particularly *kutcha* dwellings. Residents used blankets and shawls, sat in the sun and used the stove and firewood:

I use fire inside, I have an open wall at the front on the first floor so I light a fire inside there and then sleep (FGD-4)

However, using firewood and embers inside risks burns, fire and pollutant exposure.

Liquefied petroleum gas (LPG) was the predominant fuel for cooking, with just two occupants using polluting biomass fuels of wood or cow dung—outside the house. Ventilation was assessed poor in all dwellings, windows were often covered up by coolers or to prevent cold drafts (Fig. 2), and only seven dwellings had exhaust fans. Participants opened doors while cooking and used extract fans, where installed, to remove pollutants.

Participants recalled coughing due to spices from cooking and from burning cow dung. Pollutants are most likely to affect female occupants as they are responsible for cooking. Participants reported smoking “*beedi*” (organic cigarettes) indoors and the use of incense during prayers, which added to the pollution, one participant recalled: “I wake up in smoke and go to sleep in smoke” (FGD-5).

Only six households perceived good levels of lighting, eight adequate light levels, and the remaining 13 perceived lighting as poor. Most households (22 of 27) depended on electrical lighting and some reported using candles to see:

…there are no windows so sunlight does not come, so we have to light candles in the kitchens to cook… (FGD-4)

Noise levels were seen as high, with 17 households finding it noisy of which 12 reported disturbed sleep. Occupants recalled neighbours singing and talking and noted the limited privacy.

Brick walls and concrete or corrugated roofs were the most widespread materials, with some instances of red stone slabs for roofing. Corrugated roofing was used on the uppermost floor, where load-bearing was not required. Where such roofing contains asbestos, there is a potential exposure risk, especially during construction or upgrading. Usable floor areas ranged from 10.2 to 38.4 m^2^ (mean 23 m^2^) and mean occupancy of six (range 1–11 occupants); occupancy numbers were high due to a larger number of children and grandparents. Twenty-one households have less than 5 m^2^ per occupant, and 17 households have more than two occupants per room—classifying them as slum households. Lack of space meant family members had to share beds, sleep on the ground and use the floor for multiple purposes resulting in frequent cleaning to prevent contamination:

We sleep, eat and do everything on the floor, so, we clean it every day… (FGD-3)

Mosquitoes were widespread, as were open water containers (tanks, buckets, etc.) and open stagnant drains across the settlement (Fig. 2), providing an insect breeding ground. Mosquitoes were linked to illnesses—dengue and chikungunya—and reported to be particularly bothersome:

…they keep biting all day and even if you switch on the fan, they still keep biting. They bite while we cook as well. There are too many mosquitoes. (FGD-2)

Bed-nets, repellents and ceiling fans were used to repel mosquitoes, as was the burning of cow dung to produce smoke: “I burn it and that gives lots of smoke…” (FGD-1), such practices have negative impacts on air quality and health.

Pests were widely observed during household visits; mice and rats were common inside and around the dwellings. Dumping of solid waste around the settlement is widely practised, attracting rats, stray dogs and other pests. Insects were reported to bite leading to skin irritation and rashes. Rats were reported to bite while occupants were sleeping or carrying out household activities. Some households had covered drains and blocked holes, which reportedly reduced the presence of rats.

**Fig. 4:**
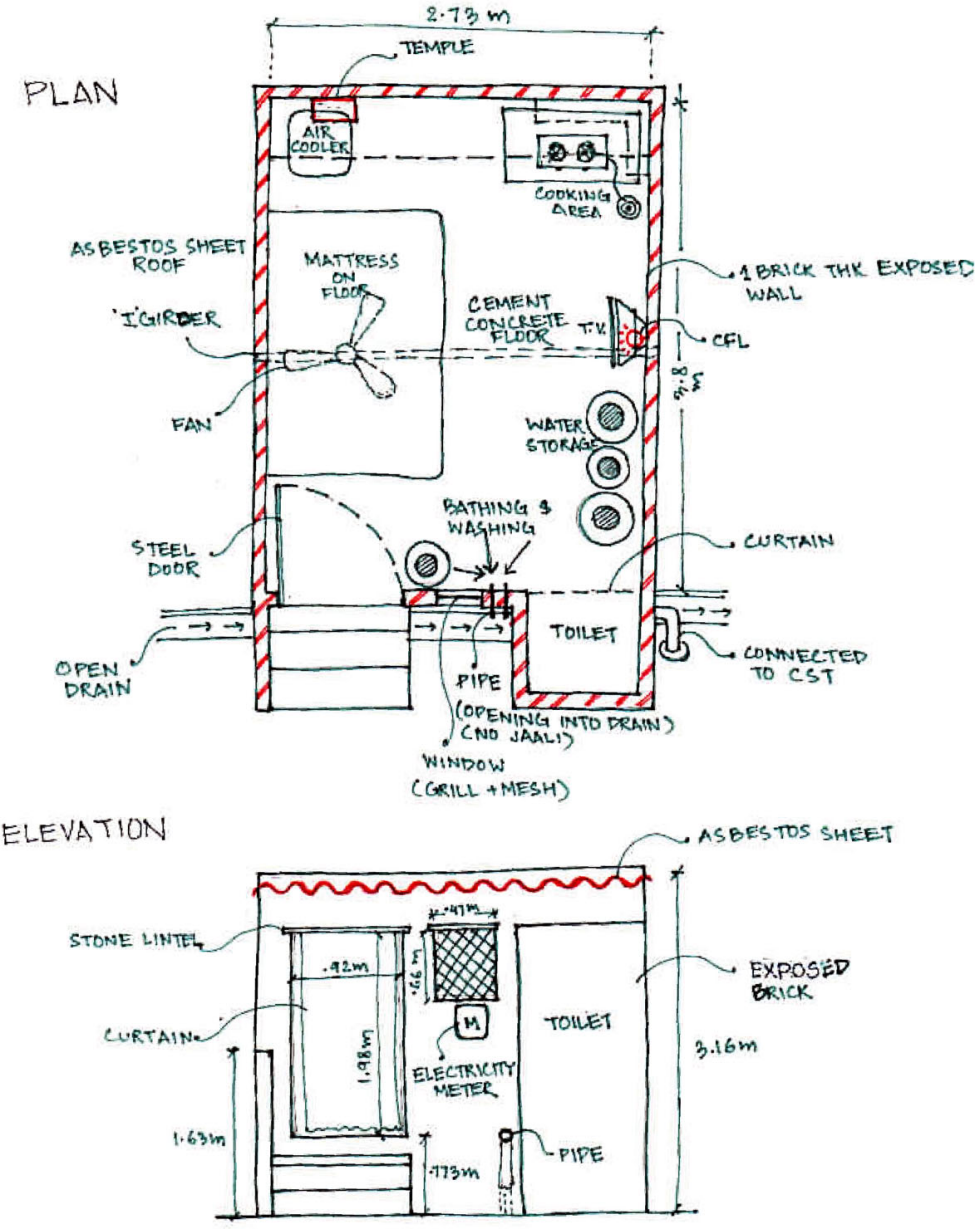
Example plan of a surveyed dwelling and multi-purpose use of indoor space

The quality of kitchen facilities, and hence domestic hygiene, was deemed poor in 14 households, adequate in four and good in nine. Many homes lacked dedicated kitchen spaces, with cooking carried out on the floor (Fig. 4). Only 12 homes had a refrigerator and cooked food was observed uncovered in pans on the floor, risking pest exposure. Participants recalled insects commonly infesting rice and wheat products, indicating improper storage. A significant amount of time was spent washing and clearing insects from infected food bags. Preservatives were added for storing foods but required washing before use.

Bathing facilities were poor in 19 houses, adequate in six houses and good in only two households, suggesting that personal hygiene is restricted. Most households lacked a separate area with bathing often taking place outside the household (11 out of 27) or within a common room, as shown in Fig. 4. Nineteen houses had toilets, with most households constructing holding tanks beneath the dwelling, which must be emptied periodically. Often the supernatant water from the tanks overflowed into drains and some toilets were situated directly over the open drains, risking significant environmental contamination. The government provided community sanitation blocks, but open defecation is widely practised. Troublingly, the lack of home toilets was reported to impact behaviour and the frequency of defecating:

… my kids are troubled. My kid is very small. She goes to the neighbour’s latrine only. If I take her outside to a community toilet, she does not go there. She does not use any other latrines and does not use a latrine for two days. She will only use the old lady’s latrine. (FGD-3)

Tankers deliver treated water daily, with all households depending on this as a source for consumption, with 20 households additionally using untreated individual or shared borewells for secondary usage (not for consumption). The collected water is stored in open containers and kept beside the dirty drains, risking contamination. Furthermore, the use of contaminated water for cleaning may lead to a significant spread of infectious diseases.

Staircases are often on the outside of the dwelling with no railing for safety. Furthermore, steps are steep, often uneven and unsuitable for those with limited mobility, such as the elderly and children. Electrical wiring quality was recorded as poor in 17 households, suggesting a risk of electrical shocks. Also, 19 households use immersion rods to heat water, which can cause electrocution if the device is not properly insulated. Cooking on open fires was carried outside resulting in fire risk, but households were observed to use retrofitted gas cylinders that pose a threat to gas leakages, fire and explosion. Cooking was often completed on the ground or in cramped spaces, heightening risks of spillages or tripping and thus burns or fires. Narrow stairways with low head clearance and shelving directly over the sleeping area present collision hazards. Structural quality was poor in the *kutcha* dwellings but good in around half the households surveyed (13 of 27), although the majority did not have columns or adequate footings as per the building standards. Households reported locking doors for safety and two households reported previous theft, suggesting intruder risk.

### Perceptions and Priorities of Hazards

The survey-based risk assessment (SBRA) and the self-assessment (SA) resulted in contrasting priorities of hazards (Table 5). The SBRA concluded heat, cold and indoor air pollution to be the biggest risks, followed by damp and mould and sanitation. Mosquitoes, domestic hygiene, food safety, asbestos and personal hygiene were also assessed to be high risk, especially in the more temporary dwelling structures (see SM). In the SA, the residents’ top-ranked hazards were damp and mould, mosquitoes, heat, pests and food infestations (see SM). Almost all households identified damp and mould and mosquitoes to be hazards, and the majority identified pests, food infestations and heat. There was no self-identification of hazards for structural collapse, falls or domestic hygiene and little identification of hazards from the water supply, sanitation, indoor air quality and personal hygiene. These differences in ranks revealed significant variations in the communities’ experiences of the hazards.

**Table 5:** Top five hazard priorities from the survey-based risk assessment, self-assessment and developed consensus

Rank	Survey-based risk assessment	Self-assessment	Consensus after focus groups
1	Heat	Damp and mould	Damp and mould
2	Cold	Mosquitoes	Heat
3	Indoor pollution	Pests	Cold
4	Damp and mould	Food infestations	Mosquitoes
5	Sanitation	Heat	Indoor pollution

**Fig. 5:**
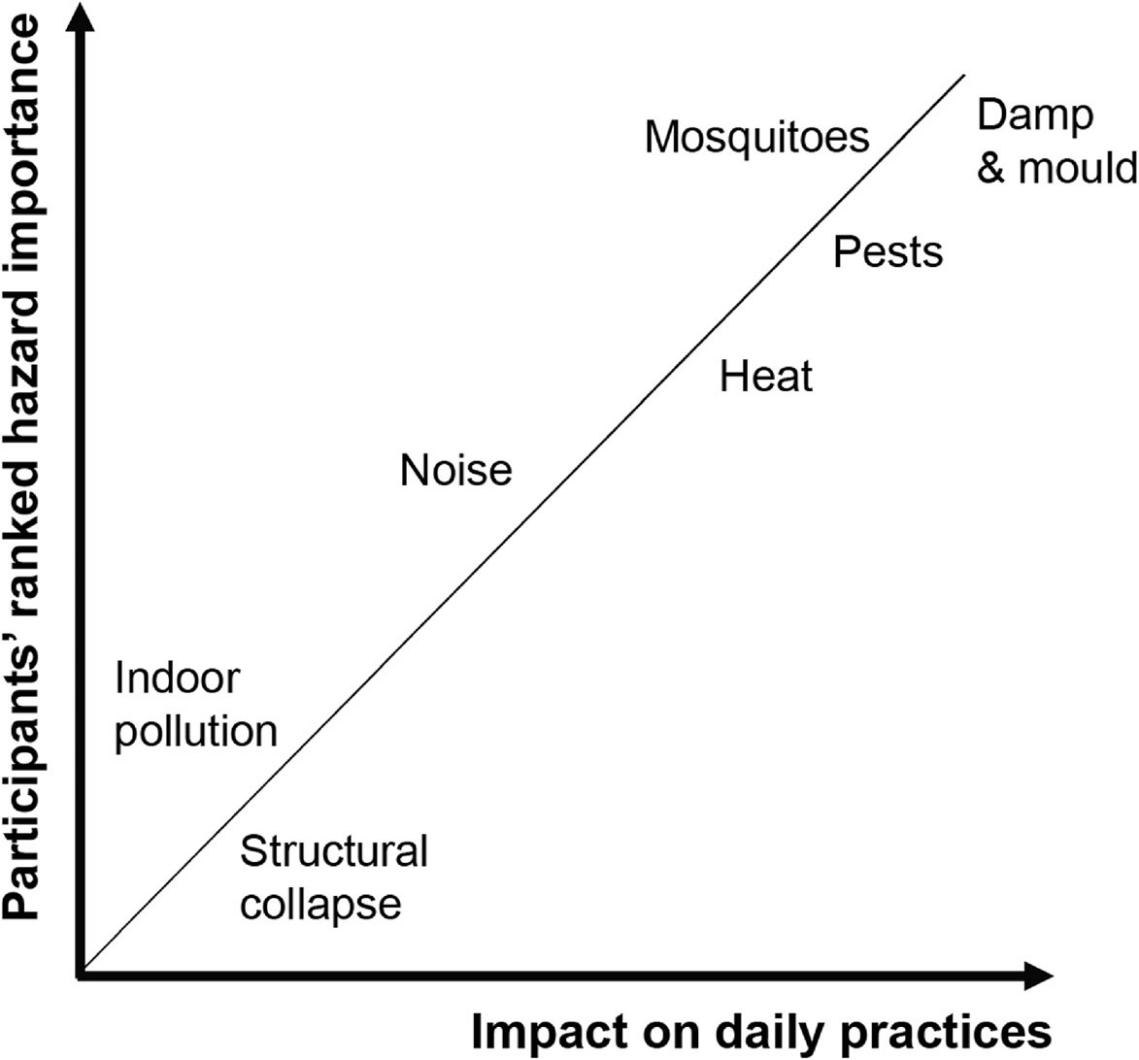
Relationship between the participants’ ranking of hazard importance and the impact of hazards of daily household practices

It appears that the lived experiences of the housing conditions were closely linked to the ranking of priorities, as hazards most highly ranked by the participants were reported to have a significant impact on daily practices; this relationship is illustrated in Fig. 5. For example, the households spent a significant amount of time dealing with issues of dampness, through the drying of belongings or the removal of water and retreating of walls. Similarly, mosquitoes, pests and food infestation were all a significant irritation to the occupants, resulting in a high ranking. Participants also recalled the wider impacts of some of the hazards as reasons for priorities:

…there are many problems because of mosquitoes. If this has been solved, people won’t fall sick and would be able to work well, be productive. Mosquitoes are the biggest problem... (FGD-3)

The SBRA evaluated the probable health outcome due to a hazard and led to a higher prioritisation of hazards that are more likely to lead to extreme harm (e.g. death, lung cancer) and was not able to consider the indirect effects and impacts on everyday practices which were of importance to the participants. This highlights the need to consider the systemic effects of housing conditions and the wider impacts on households.

However, some households considered some hazards to be outside their control, which had led to lower prioritisation:

…in the summer season it’s going to be hot, so we have accepted it… but the other problems they are man-made, the other problems, like the drainage and the mosquitoes… (FGD-5)

Similarly, for open drains, participants held the local government responsible for emptying and cleaning, so were resistant to tackling issues around poor drainage. For some hazards, households were not aware of potential solutions or how to improve conditions, indicating a lack of understanding and the need to increase awareness amongst the community.

The focus groups were used to build consensus on the prioritisation of hazards. Priorities were confirmed by asking households which hazards they would like to focus on towards the end of the session, and there was broad agreement across all typologies. It was agreed that developed inventions should address damp and mould, heat, cold, mosquitoes, indoor pollution and pests. However, it was remarked that for those “…people who do not have toilets in their house, there is a lot of distress…” (FGD-3) and so toilets should be a priority in these cases.

## Discussion

To our knowledge, this is the first in-depth investigation of housing conditions in an informal settlement that considers a wide range of housing-related health risks. We found that housing conditions do not meet requirements for good health and well-being, with significant hazards present and *kutcha* dwellings experiencing the most limiting conditions. In general, the housing investigated was unable to provide safe indoor temperatures, had poor ventilation for the removal of pollutants, experienced dampness and mould, used hazardous materials and was poorly constructed, experienced overcrowding and had poor natural lighting and little protection against noise. Furthermore, there was a substantial presence of mosquitoes, pest and food infestation, and facilities for cooking, washing and sanitation were inadequate. This illustrates the vital need for interventions to improve conditions and fulfil requirements for health. We note that the housing investigated in this work may be of higher quality than settlements built on land without tenure, where households tend not to invest in their homes due to uncertainty of removal; work should be carried out to understand the risks in these settlements.

The limiting housing conditions were revealed to impact daily practices, where households spent significant amount of time coping with hazards. This time lost could be invested elsewhere, this is likely to limit productivity and socio-economic development, hence keeping households trapped in a cycle of poverty. For example, due to illness from mosquitoes, households reported the inability to work, which restricts income and limits the ability to afford health care or invest in housing improvements. Women and girls are likely to be most impacted by the conditions, as they are responsible for most household tasks and are the most vulnerable group with little say over the household. The most illustrative example was the lack of toilets, which was reported to impact behaviour and likely to lead to the reduction of intake of food by young girls, leading to undernutrition and restricts healthy development. Inadequate sanitation is known to have significant impacts on health and well-being, in particular for females, who may experience violence or indignity through lack of access as well as by limiting drinking and eating to avoid the need to use a toilet. Improving housing conditions in these settings is therefore likely not only to improve health outcomes but also to help accelerate development and pursue gender equality.

Interventions to improve housing need a multi-sectoral approach and the involvement of different stakeholders. For example, changes to the local environment and climate could help reduce risks of heat exposure through wide-scale greening interventions to reduce local temperatures, which would require the involvement of local government. Some issues call for better governance, such as the emptying of overflowing drains, removal of refuse and provision of improved sewage and water infrastructure. Better urban planning is needed to increase green space and shading, improve ventilation between dwellings and provide sufficient living space. Capacity building and financial support are needed to improve construction practices and dwelling design, to ensure safe construction to design codes and support access to better materials and clean cooking fuels. Capacity building is also required to increase awareness of appropriate behaviours to maintain safe conditions, for example the management of wastewater, hygiene practices and the risks of smoking and use of wood for indoor heating.

We found substantial differences in the prioritisation of hazards between researchers and participants, largely due to different perspectives and lived experiences. This revealed shortcomings in conventional approaches to assess housing and health, which do not consider the impacts on practices or local perspectives. Taking these factors into account will support the development of effective interventions that are desirable for the community, which is likely to result in scalable solutions. Researchers and practitioners should ensure that participant perspectives are adequately incorporated within future methodologies to ensure the development of appropriate and effective interventions.

Although our work is limited to a single case study settlement and detailed findings are not generalisable, our methodology can be scaled to further settlements and settings to establish priorities for housing interventions. The findings and the priorities discussed in this paper have been fed into further work co-designing housing solutions with the community and experts to improve health and sustainability. We have illustrated the need to evaluate housing more holistically and to understand the impacts on daily practices. These insights have significant implications for policy-makers, the research community and practitioners, and highlight that a transdisciplinary approach is vital to develop inclusive and effective interventions to help achieve the SDGs. Further work should carry out large-scale surveys of housing conditions and health status to quantify the connections between health and housing in these settings and work with a range of stakeholders to agree on priorities and develop policies and solutions.

## Conclusions

We developed a novel transdisciplinary approach to investigate housing health hazards in participation with the community from an informal settlement in Delhi. We found that housing conditions failed to meet the requirements for health, with a substantial range of hazards present. Housing conditions were revealed to significantly affect daily practices, which in turn is likely to limit socio-economic development and gender equality. Priorities for interventions differed between the conventional survey-based approach and the participants’ self-assessment, revealing how current approaches fail to understand the multiple impacts of housing conditions and local perspectives. We recommend that future approaches assessing housing conditions involve relevant stakeholders to build consensus on priorities for intervention. Housing solutions need to consider the systemic impacts, local perspectives and, in particular, the needs of women and girls to be inclusive, effective and desirable.

## Electronic supplementary material

ESM 1 (PDF 1.04 mb)

## Footnotes

1 *Kutcha* houses are made as temporary unstable structures from plastic sheets and bamboo strips as walls and roofs on dirt floors, and *pucca* houses use more stable materials, such as brick masonry structures but are with or without reinforced columns and beams.

2 FGD-1—Kutcha dwellings, FGD-2—Semi-pucca, FGD-3—Kutcha, FGD-4—Pucca 1 and 1.5, FGD-5—Pucca 2 and 2.5, FGD-6—Pucca 2.5 and 3+
