# Supplementary material for: Evaluating Housing Health Hazards: Prevalence, Practices and Priorities in Delhi’s Informal Settlements
Source: J Urban Health. 2020 May 30;97(4):502–18. doi: 10.1007/s11524-020-00442-w (PMC7392988; doi:10.1007/s11524-020-00442-w)
Supplement: Supplementary file 1 — (PDF 1.04 mb) [file 11524_2020_442_MOESM1_ESM.pdf]

**Supplementary Material**

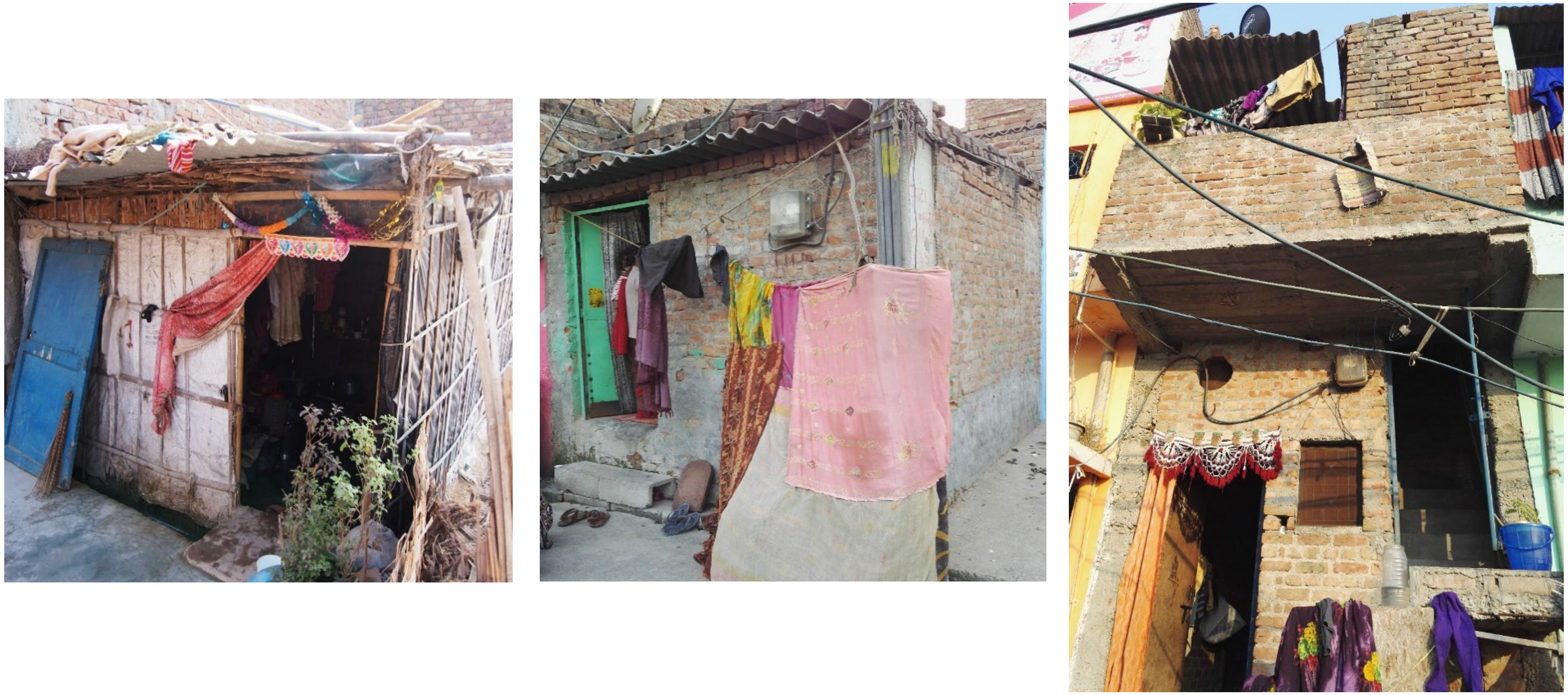

*Figure S1: Different housing typologies, from left to right: a kutcha dwelling, a semi-pucca dwelling, pucca 1.5 dwelling.*

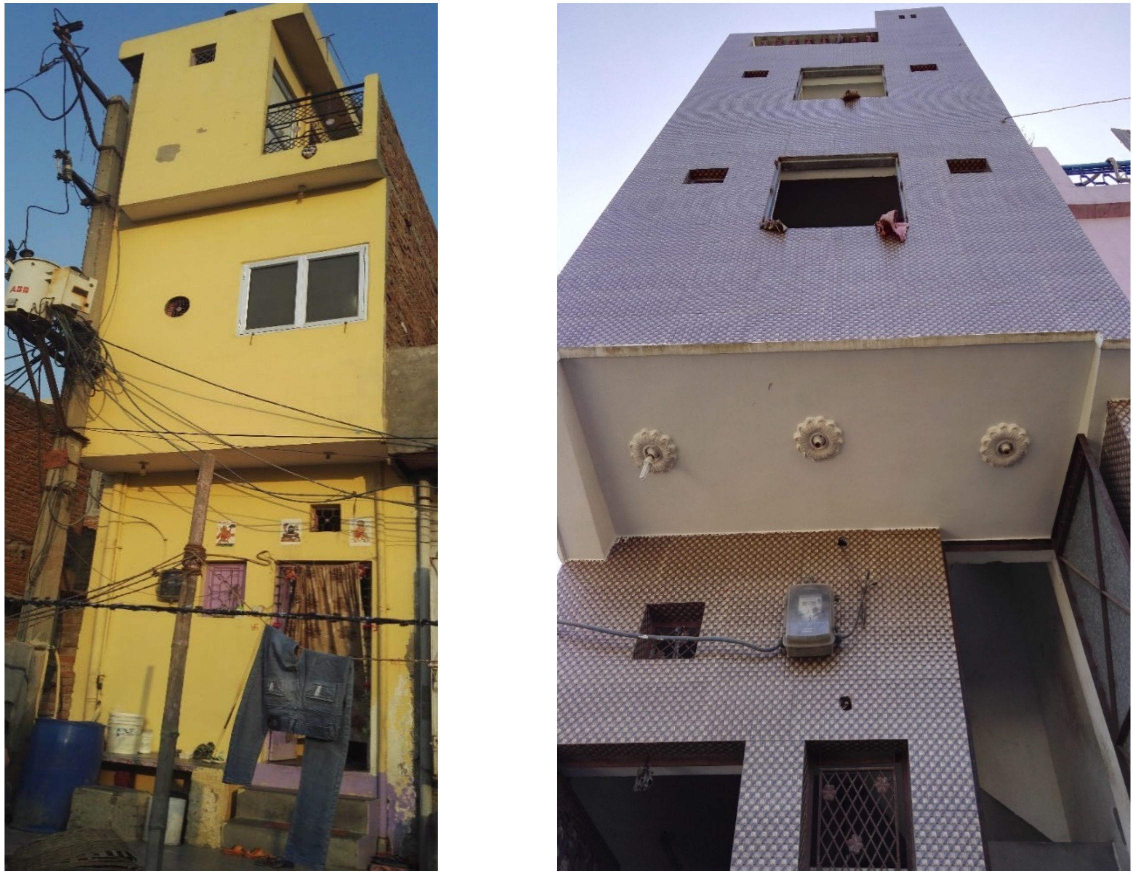

*Figure S2: Different housing typologies, from left to right: a pucca 2.5 dwelling and a pucca 3.5 dwelling.*

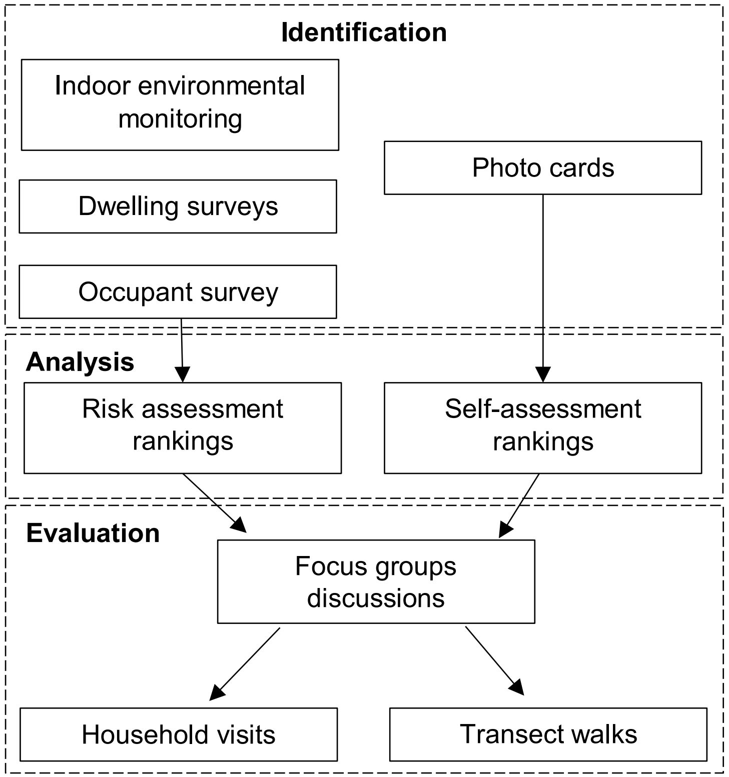


*Figure S3: Risk assessment framework and associated methods at each stage*

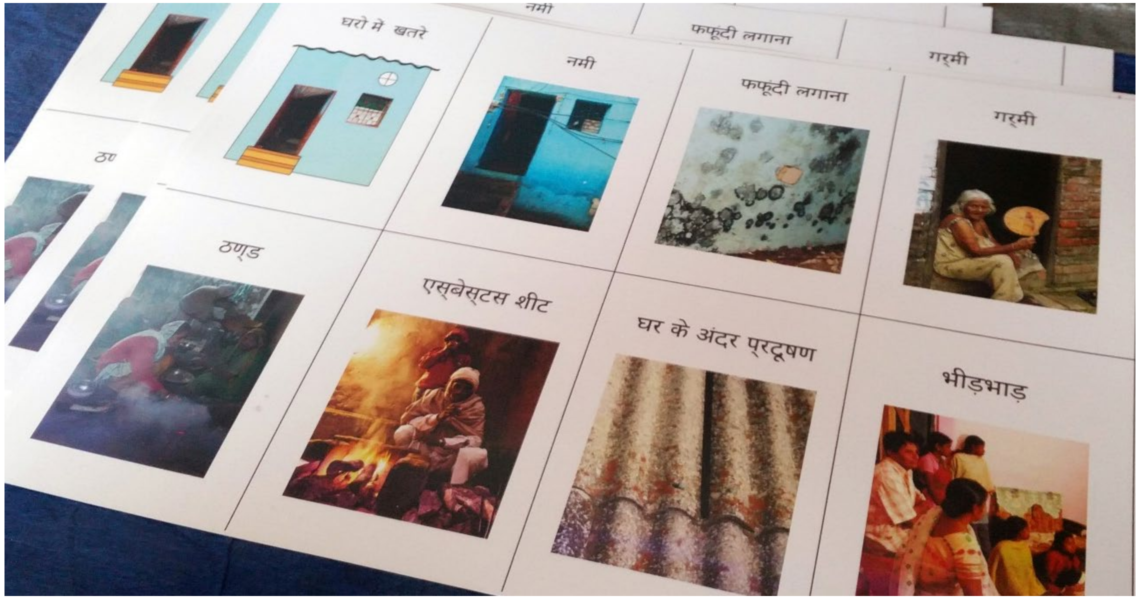


*Figure S4: Hazard picture cards used in the self-assessment*

*Table S1: Survey risk assessment results from the surveyed households, with the top five hazards highlighted in blue.*

| HH # | Typology | Damp & Mould | Heat | Cold | Indoor air pollution | Asbestos | Overcrowding | Security/Intruders | Inadequate Lighting | Noise | Mosquitoes | Domestic Hygiene | Pests | Food safety/infestation | Personal Hygiene | Sanitation | Water Supply | Falls | Electrical Shocks | Flames, fire, burns | Collisions | Structural collapse |
|---|---|---|---|---|---|---|---|---|---|---|---|---|---|---|---|---|---|---|---|---|---|---|
| A | Kutcha | 12 | 16 | 16 | 16 | 3 | 8 | 4 | 8 | 4 | 12 | 8 | 8 | 8 | 8 | 16 | 16 | 8 | 8 | 12 | 4 | 12 |
| B | Kutcha | 12 | 16 | 16 | 16 | 3 | 8 | 4 | 8 | 4 | 9 | 8 | 8 | 8 | 4 | 8 | 16 | 4 | 8 | 9 | 2 | 12 |
| C | Kutcha | 12 | 16 | 16 | 16 | 12 | 8 | 2 | 4 | 4 | 12 | 8 | 8 | 8 | 8 | 16 | 16 | 4 | 8 | 12 | 1 | 12 |
| D | Kutcha | 12 | 16 | 16 | 16 | 12 | 6 | 4 | 8 | 4 | 12 | 8 | 8 | 8 | 8 | 16 | 16 | 4 | 8 | 12 | 1 | 12 |
| E | Semi-pucca | 12 | 16 | 16 | 4 | 12 | 2 | 1 | 4 | 4 | 3 | 2 | 6 | 6 | 2 | 4 | 4 | 4 | 6 | 3 | 2 | 3 |
| F | Semi-pucca | 12 | 16 | 16 | 16 | 12 | 8 | 1 | 8 | 4 | 12 | 8 | 8 | 8 | 8 | 16 | 16 | 8 | 8 | 9 | 3 | 12 |
| G | Semi-pucca | 3 | 12 | 16 | 16 | 12 | 4 | 1 | 8 | 2 | 12 | 8 | 4 | 4 | 8 | 16 | 12 | 2 | 6 | 12 | 1 | 6 |
| H | Semi-pucca | 12 | 16 | 12 | 16 | 12 | 6 | 1 | 2 | 2 | 12 | 8 | 2 | 2 | 8 | 16 | 8 | 2 | 8 | 9 | 2 | 6 |
| I | Semi-pucca | 12 | 16 | 16 | 16 | 6 | 4 | 4 | 8 | 4 | 9 | 6 | 8 | 8 | 8 | 16 | 16 | 2 | 8 | 12 | 1 | 12 |
| J | Pucca1 | 3 | 8 | 4 | 12 | 3 | 8 | 3 | 4 | 2 | 9 | 4 | 2 | 2 | 6 | 12 | 8 | 2 | 4 | 3 | 1 | 3 |
| K | Pucca1 | 3 | 4 | 8 | 16 | 3 | 8 | 1 | 4 | 2 | 12 | 4 | 4 | 4 | 8 | 16 | 16 | 6 | 6 | 6 | 4 | 3 |
| L | Pucca1.5 | 6 | 8 | 12 | 12 | 12 | 2 | 1 | 4 | 1 | 9 | 4 | 4 | 4 | 8 | 16 | 4 | 8 | 6 | 9 | 4 | 6 |
| M | Pucca1.5 | 12 | 12 | 12 | 8 | 9 | 4 | 1 | 4 | 2 | 6 | 2 | 6 | 6 | 6 | 12 | 8 | 2 | 4 | 9 | 1 | 3 |
| N | Pucca1.5 | 12 | 16 | 8 | 4 | 9 | 8 | 1 | 2 | 1 | 9 | 2 | 6 | 6 | 2 | 4 | 8 | 6 | 6 | 3 | 2 | 9 |
| O | Pucca1.5 | 3 | 16 | 16 | 16 | 6 | 8 | 4 | 4 | 3 | 12 | 6 | 6 | 6 | 4 | 8 | 8 | 6 | 8 | 12 | 3 | 9 |
| P | Pucca1.5 | 12 | 16 | 16 | 16 | 12 | 2 | 4 | 8 | 4 | 9 | 4 | 2 | 2 | 4 | 8 | 4 | 6 | 6 | 12 | 4 | 6 |
| Q | Pucca1.5 | 12 | 16 | 16 | 12 | 12 | 2 | 3 | 8 | 3 | 12 | 8 | 8 | 8 | 8 | 16 | 16 | 8 | 2 | 3 | 4 | 3 |
| R | Pucca2 | 9 | 4 | 8 | 4 | 3 | 2 | 1 | 2 | 1 | 3 | 2 | 2 | 2 | 2 | 4 | 4 | 4 | 4 | 3 | 1 | 3 |
| S | Pucca2 | 9 | 12 | 12 | 16 | 3 | 8 | 1 | 8 | 1 | 3 | 4 | 6 | 6 | 4 | 8 | 4 | 4 | 8 | 12 | 2 | 6 |
| T | Pucca2 | 12 | 16 | 16 | 16 | 3 | 6 | 2 | 6 | 3 | 3 | 6 | 8 | 8 | 4 | 8 | 4 | 8 | 2 | 12 | 4 | 3 |
| U | Pucca2 | 12 | 16 | 16 | 12 | 3 | 6 | 1 | 4 | 4 | 6 | 2 | 8 | 8 | 2 | 4 | 4 | 4 | 2 | 9 | 1 | 3 |
| V | Pucca2* | 12 | 12 | 16 | 8 | 3 | 2 | 1 | 6 | 1 | 6 | 4 | 2 | 2 | 4 | 8 | 4 | 2 | 2 | 3 | 1 | 3 |
| W | Pucca2.5 | 9 | 12 | 12 | 4 | 9 | 2 | 1 | 4 | 2 | 6 | 4 | 2 | 2 | 6 | 12 | 4 | 8 | 2 | 3 | 3 | 3 |
| X | Pucca2.5 | 6 | 4 | 4 | 4 | 3 | 2 | 2 | 2 | 2 | 6 | 2 | 4 | 4 | 2 | 4 | 8 | 6 | 4 | 3 | 2 | 3 |
| Y | Pucca3+ | 12 | 16 | 16 | 4 | 6 | 2 | 1 | 4 | 1 | 3 | 2 | 2 | 2 | 2 | 4 | 4 | 2 | 2 | 3 | 1 | 3 |
| Z | Pucca3+ | 3 | 4 | 8 | 4 | 3 | 8 | 1 | 2 | 1 | 3 | 2 | 2 | 2 | 2 | 4 | 4 | 6 | 2 | 3 | 1 | 3 |
| AA | Pucca3+ | 6 | 4 | 4 | 4 | 3 | 2 | 1 | 2 | 1 | 6 | 4 | 2 | 2 | 6 | 12 | 4 | 6 | 2 | 9 | 3 | 3 |

*Table S2: Self-assessment results from the surveyed households, with the top five hazards highlighted in blue.*

| HH # | Typology | Damp & Mould | Heat | Cold | Indoor air pollution | Asbestos | Overcrowding | Security/Intruders | Inadequate Lighting | Noise | Mosquitoes | Domestic Hygiene | Pests | Food safety/infestation | Personal Hygiene | Sanitation | Water Supply | Falls | Electrical Shocks | Flames, fire, burns | Collisions | Structural collapse |
|---|---|---|---|---|---|---|---|---|---|---|---|---|---|---|---|---|---|---|---|---|---|---|
| A | Kutcha | 2 | - | - | 11 | - | - | 8 | 10 | - | 6 | - | 4 | 5 | 9 | - | - | - | 3 | 7 | 1 | - |
| B | Kutcha | 1 | 9 | 8 | 10 | - | - | - | 7 | - | 3 | - | 2 | 6 | - | - | - | - | 4 | 5 | - | - |
| C | Kutcha | 1 | 10 | - | 11 | 9 | - | 8 | 3 | 6 | 2 | - | 7 | 4 | 5 | - | - | - | - | - | 12 | - |
| D | Kutcha | 1 | 2 | - | - | - | 7 | - | - | 6 | 5 | - | - | 4 | 8 | - | - | - | 3 | - | - | - |
| E | Semi-pucca | 1 | - | - | - | - | - |  | - | - | 2 | - | - | - | - | - | - | - | 3 | - | - | - |
| F | Semi-pucca | 3 | 8 |  | 2 | 9 | 5 | - | - | - | 6 | - | 7 | 1 | 4 |  | - | - | - | - | - | - |
| G | Semi-pucca | 2 | 1 | - | - | 3 | - | - | - | - | 5 | - | - | - | - | - | 4 | - | - | - | - | - |
| H | Semi-pucca | 3 | 2 | - | - | 5 | - | - | - | - | 1 | - | - | 4 | - | - | - | - | - | - | - | - |
| I | Semi-pucca | 1 | 5 | 6 | 9 | 8 | - | - | - | - | 2 | - | - | 7 | - | - | - | - | 3 | 4 | 10 | - |
| J | Pucca1 | 2 | 1 | - | - | - | 3 | - | - | - | 5 | - | 4 | - | - | - | - | - | - | - | - | - |
| K | Pucca1 | 1 | - | - | - | - | - | - | 5 | - | 2 | - | 3 | 4 | - | - | - | - | - | - | - | - |
| L | Pucca1.5 | 1 | - | - | - | - | - | - | - | - | 5 | - | 3 | 4 | - | - | - | - | - | 2 | - | - |
| M | Pucca1.5 | 1 | 5 | - | - | - | - | - | - | - | 3 | - | - | 4 | - | - | - | - | 6 | - | 2 | - |
| N | Pucca1.5 | 5 | 6 | - | - | - | - | - | - | - | 2 | - | 1 | 3 | - | - | - | - | 4 | - | - | - |
| O | Pucca1.5 | 4 | 1 | - | - | - | - | - | 5 | - | - | - | 2 | 3 | 6 | - | - | - | - | - | - | - |
| P | Pucca1.5 | 1 | - | - | - | - | - | - | 2 | - | 3 | - | 4 | - | - | - | - | - | 5 | - | - | - |
| Q | Pucca1.5 | 1 | 3 | - | - | - | 4 | 6 | - | 5 | 2 | - | 7 | 8 | - | - | - | - | 9 | - | - | - |
| R | Pucca2 | 1 | - | - | - | - | - | - | - | - | 2 | - | - | - | - | - | - | - | - | - | - | - |
| S | Pucca2 | 1 | - | - | - | - | - | - | 4 | - | 2 | - | 3 | 5 | 6 | 7 | - | - | - | - | - | - |
| T | Pucca2 | - | - | 5 | 6 | - | 3 | - | - | - | 2 | - | 1 | 4 | - | - | - | - | - | - | - | - |
| U | Pucca2 | 3 | 1 | - | - | - | 4 | 5 | - | - | 2 | - | - | - | - | - | - | - | - | - | - | - |
| V | Pucca2* | 1 | - | 3 | - | - | - | - | 4 | - | 2 | - | - | - | - | - | - | - | - | - | - | - |
| W | Pucca2.5 | 1 | - | - | - | - | 2 | 3 | - | 8 | 7 | - | - | - | 4 | - | - | - | 6 | 5 | 9 | - |
| X | Pucca2.5 | 1 | - | - | - | - | 5 | - | - | - | 2 | - | 3 | - | 4 | - | - | - | - | - | - | - |
| Y | Pucca3+ | - | 1 | - | 2 | - | 4 | - | - | 5 | - | - | - | - | - | 3 | - | - | - | - | - | - |
| Z | Pucca3+ | - | 4 | - | - | - | 1 | - | 5 | 2 | 3 | - | - | - | - | - | - | - | - | - | - | - |
| AA | Pucca3+ | 1 | - | - | - | - | - | - | 6 | - | 2 | - | 3 | 4 | - | 5 | - | - | - | - | - | - |
